# The relation between clinically diagnosed and parent-reported feeding difficulties in children with and without clefts

**DOI:** 10.1007/s00431-023-04852-1

**Published:** 2023-03-02

**Authors:** Iris A. C. de Vries, Camille H. A. L. Guillaume, Wouter M. Penris, Anne Merijn Eligh, Rene M. J. C. Eijkemans, Moshe Kon, Corstiaan C. Breugem, Marijn W. G. van Dijk

**Affiliations:** 1 Department of Paediatric Plastic Surgery, Wilhelmina Children’s Hospital, University Medical Centre, PO Box 85500, 3508 GA Utrecht, the Netherlands; 2 Department of Paediatric Plastic Surgery, Emma Children’s Hospital, University of Amsterdam, Amsterdam, the Netherlands; 3 Faculty of Behavioral and Social Sciences/Applied Linguistics, University of Groningen, Groningen, the Netherlands; 4 Department of Biostatistics and Research Support, Julius Centre, University Medical Centre, Utrecht, the Netherlands; 5 Heymans Institute for Psychological Research, University of Groningen, Groningen, the Netherlands

**Keywords:** Cleft palate, Feeding difficulties, Feeding scale, Spoon feeding, Oral motor assessment

## Abstract

A cleft lip and/or palate (CL/P) is one of the most common craniofacial malformations, occurring worldwide in about one in 600–1000 newborn infants. CL/P is known to influence the feeding process negatively, causing feeding difficulties in 25–73% of all children with CL/P. Because there is a risk for serious complications in these children regarding feeding difficulties, there is often a need for intensive medical counseling and treatment. At this moment, adequate diagnosis and measurement remain a challenge and often lead to a delayed referral for professional help. Since parents play a big part in reporting feeding difficulties, it is important to help objectify parents’ experiences, as well as the use of a frontline screening instrument for routine check-ups during medical appointments. The aim of this study is to investigate the relationship between parent perspective and standardized observation by medical professionals on feeding difficulties in 60 children with and without clefts at the age of 17 months. We focus on the information from parents and health professionals by comparing the Observation List Spoon Feeding and the Schedule for Oral Motor Assessment with the validated Dutch translation of the Montreal Children’s Hospital Feeding Scale.

*  Conclusion*: There is a need for timely and adequate diagnosis and referral when it comes to feeding difficulties in children with CL/P. This study underscores the importance of combining both parental observations and measurements of oral motor skills by healthcare professionals to enable this.

**Table:** 

**What is Known:**
• *Early identification of feeding difficulties can prevent adversely affected growth and development.*
• *Clefts increase the probability of feeding difficulties; however, the diagnostic trajectory is unclear.*
• *The Observation List Spoon Feeding (OSF) and Schedule for Oral Motor Assessment (SOMA) are validated to measure oral motor skills. The Montreal Children’s Hospital Feeding Scale Dutch version (MCH-FSD) has been validated for the parental perception of infant feeding difficulties.*
**What is New:**
• *Parents of children with CL/P experience relatively few feeding problems in their child on average.*
• *Oral motor skills for spoon feeding are associated with oral motor skills for solid foods in children with CL/P.*
• *The extent of the cleft is associated with experiencing more feeding difficulties in children with CL/P.*

## Introduction

A cleft lip and/or palate (CL/P) is a common craniofacial malformation, occurring worldwide in about one in 600–1000 newborn infants [1–3]. Facial clefts are known to influence the feeding process negatively [4–7] and cause feeding difficulties in 25–73% of all children with CL/P [4, 6, 8–10]. Feeding difficulties occur particularly often in children with cleft of the palate only [6, 7, 11].

It is known that feeding difficulties occur frequently in healthy children as well; they are reported in 25–50%, depending on the definition used [12]. Severe feeding difficulties are estimated to occur in 3–10% of children. In 2019, Goday et al. proposed a consensus definition for pediatric feeding difficulties which is now used for diagnostic purposes in the ICD-11 [13]. However, at this moment, there is no universally accepted consensus about the exact definition of feeding difficulties and what constitutes them. This complicates adequate diagnosis and measurement and—as a consequence—timely referral to professional help.

Intensive medical counseling and treatment are often required regarding feeding difficulties in children with CL/P because of the risk for serious complications [2, 6, 14–16]. For this reason, it has been argued that there is a need for a frontline screening instrument which can be used for routine check-ups during medical appointments [17]. Besides this, a parental questionnaire can be used to help objectify parents’ experiences. A reliable and validated parental report instrument is the Montreal Children’s Hospital Feeding Scale (MCH-FS) [12]. This questionnaire contains only 14 items, is validated for French, English, and Dutch children, and has been demonstrated to have good sensitivity and specificity. Additionally, to measure feeding difficulties from the oral motor perspective, a few reliable and validated instruments have been developed, such as Observation List Spoon Feeding (OSF) [18] and Schedule for Oral Motor Assessment (SOMA) [19]. We argue that comparing the parent perspective with the standardized observation by medical professionals might enable early detection and treatment of feeding difficulties in these children. Therefore, the present study concentrates on information from parents and health professionals by comparing the OSF and SOMA with the validated Dutch translation of the MCH-FS (MCH-FSD, or in Dutch: SEP) [20].

The research questions of the present study are: (1a) what is the prevalence of parentally-reported feeding difficulties (MCH-FSD) in children with CL/P, as compared to children without CL/P in the general population, and (1b) is there a difference in symptomatic patterns between both groups; (2) in children with CL/P, is there a correlation between parentally-reported feeding problems and oral motor skills as reported by a speech therapist; and (3) what is the relation between CL/P patient characteristics (cleft severity, cleft type, and age of surgery), parentally reported feeding difficulties, and oral motor skills.

## Methods

### Participants

By analyzing the medical records of consecutively treated children in Wilhelmina Children’s Hospital (WCH), Utrecht, the Netherlands, 60 children (29 girls) born with CL/P were included at the age of 17 months (mean 74.45 weeks of age, SD 1.16). See Table 1 for the patient’s characteristics.

**Table 1:** Demographic data and participant characteristics of the study population

**CHARACTERISTICS**		**TOTAL (**N** = 60)**
**AGE (WEEKS)**		
** MEAN (± SD)**^**a**^	59	74.45 (± 1.16)
**LENGTH (CM)**		
** MEAN (± SD)**^**a**^	58	81.44 (± 2.93)
**WEIGHT (KG)**		
** MEAN (± SD)**^**a**^	57	10.88 (± 112)
**SEX**		
**N, (%)**	Female	29 (48.3%)
	Male	31 (51.7%)
**CLEFT TYPE**^**b**^		
** N, (%)**	CLP	
	Unilateral	29
	Bilateral	13
	CP	18
**TIMING OF SURGERY**	Early	30 (50.0%)
** N, (%)**	Late	30 (50.0%)

^a^*SD* standard deviation

^b^*CL* cleft lip, *CLP* cleft lip and palate, *CP* cleft palate only

Data from the children were obtained from a WCH-based study, which was approved by the Medical Ethics Board of the Utrecht University Medical Centre, the Netherlands (protocol no.: 11–340/K). Exclusion criteria were cleft lip only, adoption, and inadequate understanding of the Dutch language by parents. For the WHC-based study, data was derived at four timepoints during visits at the ages of 6, 9, 13, and 17 months. For our study, we analyzed the data obtained at 17 months, because we hypothesized that the chance of developing proper oral motor skills was more equal for both groups a few months after surgical closure of the palate. All data used in our study is conducted post-repair.

This dataset was compared with a control group sample of 98 children in the general population with roughly the same age range (53 girls, mean age 76.46 weeks). This control-group sample originated from a larger database (*n* = 1448), which had a 16% response rate within children between 6 months and 4 years of age [20]. The selection criterium for the control group was an age range between 69 and 79 weeks.

### Measures

#### OSF

The OSF measures the qualitative and quantitative aspects of the development of oral motor skills [18]. The OSF is filled out by a professional speech therapist, who observes a video in which five assisted spoonsful are administered by one of the parents. Seven different items are scored regarding successful oral motor behavior (OSF items 11–17). Hence, the maximum score for the OSF is 35 points (quantitative aspect), indicating full control of the skill of spoon feeding. Next, the professional scores six different items for dysfunctional behavior (OSF items 8–13), to obtain a general impression of possible dysfunctional oral motor/sensory behavior during spoon feeding (qualitative aspect). Normal development is assumed when children take an average of 5.7 weeks (SD 2.1) to develop and master the skill of spoon feeding. Abnormal development is assumed when children score 2 SD above the average (9.9 weeks).

#### SOMA

The SOMA measures the development of oral motor skills [19]. A speech therapist observes a video of the infant’s oral motor skills during the parental administration of a solid food substance and scores the SOMA, which is an observational list consisting of five different categories: cup, trainer cup, solids, bottle, and cracker. The lists for “solids” and “cracker” were used in this study, as the consistency of those foods is appropriate for the age of 17 months. For the solids and cracker categories, a score above respectively 4 and 9 indicated oral motor dysfunction. Reilly et al. 1995 report excellent levels of interrater reliability (kappa values between 1 and 0.75) for the presence/absence of 69% of discrete oral-motor behaviors and test–retest reliability (for 85% of ratable behaviors) [22]. The SOMA has a positive predictive value greater than 90% and a sensitivity greater than 85% for the detection of oral motor dysfunction in infants [22].

#### MCH-FSD

The MCH-FSD has the primary aim of quickly identifying feeding difficulties from a parental perspective in children aged between 6 and 36 months. Ramsay et al. [12] report excellent validity between clinical and normative samples and excellent test–retest reliability (normative *r* = 0.845, clinical *r* = .92). Identical to the MCH-FS, the Dutch version (MCH-FSD) consists of 14 items that measure specific domains, namely oral motor, oral sensory, (lack of) appetite, parental worry, behavior at the table, feeding strategies, and family responses to feeding. The MCH-FSD can be used with children who have started eating solid foods. The minimum score is 14, indicating no feeding difficulties, while the maximum score is 98 points. This score is compared with the average for children in the same age category.

### Procedure

Children with a CL/P visited the hospital (WCH group) at the age of 17 months, and all data were collected between 2012 and 2017. Parents were asked to fill out the MCH-FSD, after which a video was made of the feeding process and the recording was scored by a speech therapist, using the OSF and the SOMA. This approach was chosen to ensure a minimal amount of stress for both the parents and the child and to prevent observer expectancy.

The control group data were obtained in collaboration with the Groningen Municipal Health Services (Gemeentelijk Gezondsheidsdienst Groningen, GGD), a local health authority who manage the regional Infant Welfare Centers (IWCs). Parents were given full disclosure during routine check-ups of their children, and informed consent was obtained.

A total of 10,000 MCH-FSD questionnaires were sent to parents of children aged 6 months to 4 years who had visited the IWC between January and March 2011. The MCH-FSD was filled out anonymously and could be returned by mail to the Department of Developmental Psychology at the University of Groningen. Alternatively, parents could also fill out the MCH-FSD while they were at the IWC. All resulting data were collected at the University of Groningen, and the data-collection process was approved by the Ethical Committee of Psychology (number ppo-010–033).

### Analytic plan

In order to answer the first research question regarding the prevalence of parentally reported feeding difficulties (MCH-FSD), an independent samples T-test was performed to test whether children with CL/P have a higher total score on the MCH-FSD as compared to children in the control group, employing an alpha of .05. Likewise, to discover potential symptomatic differences between groups, the individual items of the MCH-FS were compared using independent samples T-tests, with alpha = .05. For each T-test, Levene’s test was used to determine equality of variance and Shapiro Wilke’s test for normality. Based on this alpha, earlier-given sample sizes, and a predicted effect size of .5, a post-hoc power analysis in G*Power showed that the resulting power (1–ß) is sufficient at .858.

To clarify if there was an association between feeding difficulties as measured by the OSF, SOMA, and MCH-FSD in the CL/P group, Pearson correlations were computed, with an alpha of .05 each. A post-hoc power analysis (bivariate normal model) with a predicted r of .30, earlier-given sample sizes, and alpha indicated a somewhat low power (1–ß) at .756.

Investigating the relation between the scores for all instruments and patient characteristics, multivariate analysis of variance (MANOVA) was employed. The dependent variables were the total scores from the MCH-FSD, OSF, SOMA-solid, and SOMA-cracker. Involvement of the lip regarding the cleft (yes/no), the type of cleft (5 types), and the age of surgery (either at 6 of 12 months of age) were independent variables. A post-hoc power analysis was performed: a MANOVA, with global effects, predicted effect size of .25, 4 groups, and 4 response variables, resulted in a more than sufficient estimated power (1–ß) at .997.

## Results

For the prevalence of feeding problems as measured by the MCH-FSD, the independent samples t-test revealed a significant difference in feeding problems between the control and intervention group (t(156.0) = 4.136; *p* < .001) where the CL/P group showed a lower score (*M* = 23.57; *SD* = 6.95) as compared to the control group (*M* = 29.40; *SD* = 9.46). The equal variance was assumed as Levene’s test did not reach significance (*p* = .076). As anticipated, Shapiro Wilk’s test showed there was no normal distribution of the MCH total scores in either the control (*p* < .001) or intervention group (*p* = .007).

Equality of variance was assumed for MCH items 3–6, 8–11, and 14 with Levene’s test results ranging between *p* = .070 and *p* = .458. Conversely, for items 1, 2, 7, and 13 eq. of variance was not assumed with values between *p* = .001 and *p* = .009. Table 2 outlines the results of the sample scores at the level of the individual items, for six items significant differences were revealed: item 1 (t(156.0) = 4.359, *p* < .001), item 2 (t(153.8) = 2.842, *p* = .005), item 3 (t(156.0) = 3.161, *p* = .002), item 5 (t = (156.0)2.340, *p* = .021), item 11, (t(156.0) =  −2.577, *p* = .011), and item 13, (t(140.12) = 2.003, *p* = .047). However, these differences were very small, and in all items, the CL/P sample indicated fewer problems, except for item 11, where the CL/P group scored higher; this item rates the child’s chewing and/or sucking abilities (MCH-11).

**Table 2:** Mean differences in MCH item scores between the CL/P and Control group, with significance

Item	**CL/P** mean/SD	**mean/SD** mean/SD	Mean difference	Sig. (2-tailed)	Item text
1	1.75/.75	2.44/1.22	−.69	< .001**	How do you find mealtimes with your child?
2	1.42/.87	1.90/1.26	.48	.005*	How worried are you about your child’s eating?
3	2.30/1.33	2.96/1.24	−.659	.002*	How much appetite (hunger) does your child have?
4	5.70/1.81	5.11/1.92	−.588	.058	When does your child start refusing to eat during mealtimes?
5	2.30/.79	2.63/.91	.333	.021*	How long do mealtimes take for your child (in minutes)?
6	2.08/1.24	2.44/1.36	.355	.101	How does your child behave during mealtimes?
7	1.33/.63	1.49/.87	.157	.195	Does your child gag or spit or vomit with certain types of food?
8	1.73/1.06	1.83/1.32	−.093	.644	Does your child hold food in his/her mouth without swallowing it?
9	1.87/1.40	2.21/1.64	.348	.174	Do you have to follow your child around or use distractions (toys, TV) so that your child will eat?
10	1.70/1.03	1.81/1.23	−1.106	.578	Do you have to force your child to eat or drink?
11	2.27/1.26	1.79/1.06	−.481	.011*	How are your child’s chewing (or sucking) abilities?
12	1.93/1.58	1.52/1.17	−.413	.083	How do you find your child’s growth?
13	1.30//.74	1.56/.87	−.261	.047*	How does your child’s feeding influence your relationship with him/her?
14	1.58/1.28	1.94/1.46	.355	.123	How does your child’s feeding influence your family relationships?

*Difference is significant at the .05 level (2-tailed)

**Difference is significant at the .01 level (2-tailed)

Table 3 outlines the association between parentally reported feeding problems and oral motor skills. When comparing the MCH-FSD with the OSF and SOMA respectively, very weak correlations (between *r* =  −.028 and *r* = .151) were found, and these did not reach significance (*p* > .127 or higher). In contrast, a strong negative association was found between the OSF and SOMA-solids, *r* =  −.565 (*p* < .001). Between the OSF and SOMA-cracker, a weak negative relation was found, *r* =  −.259 (*p* = .024). The results of all Pearson correlation analyses are presented in Table 3. Normality was violated with Shapiro Wilk’s reaching significance for each dependent value (*p* <  = .006). However, correlation significances and strengths were equally visible when using Spearman-Rho, the non-parametric equivalent.

**Table 3:** Pearson correlations between the MCH-FSD, OSF, and SOMA

	MCH-FSD	OSF	SOMA-solids
OSF score	.013		
SOMA-solids	−.028	−.565^**^	
SOMA-cracker	.151	−.259^*^	.255^*^

Finally, for the relation of patient characteristics and feeding problems (MCH-FSD) or skill (OSF, SOMA), the three-way MANOVA was executed. Box’ M test of equality of covariance was not significant (F(30) = 1.297, *p* = .132) and Levene’s normality test was significant for the MCH-FS (F(15,39) = 1.942, *p* = .049), OSF (F(15,39) = 6.557, *p* < .001), and SOMA-solids (F(15,39) = 3.820, *p* < .001). The MANOVA revealed that there was no significant interaction effect between the type of cleft, cleft severity, and age of surgery on the combined dependent variables MCH-FDS, OSF, SOMA-solids, SOMA-cracker (*p* = .171), nor did any of the interactions between the independent variables have a significant interaction effect on them. The only significant effect observed on the dependent variables was the type of cleft, (F(156.0) = 2.104, *p* = .011; Pillai’s Trace = .710). Mean scores for the types are shown in Table 4.

**Table 4:** Mean scores and standard errors for cleft severity

Cleft type	MCH-FSD/SE	OSF/SE	SOMA-solids/SE	SOMA-cracker/SE
Type 0	17.500/4.221	35.000/3.178	2.000/.582	−8*10^−16^/1.269
Type 1	27.667/3.146	31.000/2.369	3.000/.433	.833/.946
Type 2	25.536/1.475	33.759/1.110	1.815/.203	.339/.443
Type 3	28.567/1.848	31.050/1.391	.742/.255	.575/.556
Type 4	18.611/1.977	33.766/1.488	.1462/.272	.889/.594

## Discussion

The goal of the present paper was to investigate the usefulness of a standardized screening instrument, the MCH-FSD, for the early detection of feeding problems, based on parental report, to allow health professionals to objectively interpret and address these parentally-reported feeding problems at an early stage and relate them to the level of oral motor skills and use both to enable adequate treatment of feeding difficulties in these children.

Currently, feeding difficulties are explained using a bio-psychological-social model [22, 23]. Therefore, when analyzing feeding difficulties, it is imperative to measure not only the oral motor skills but also the parent’s perspective and therefore the psychological and social signals that might indicate feeding difficulties. Early detection and treatment in children with CL/P are important because of the risk of serious complications. Because of inadequate separation between the oral and nasal cavity during feeding [2, 14], excessive air intake and nasal regurgitation can occur [2], leading to an increased risk of choking while feeding [6, 15] and possibly to aspiration and pulmonary complications [16]. Subsequently, next to severe dehydration [24], feeding difficulties can result in impaired growth [15, 25, 26] and failure to thrive. As a result, this is causing stress, anxiety, and frustration in parents during the feeding process [2, 27–33]. It has been reported that feeding difficulties in early childhood can also negatively influence maternal attachment [34], mental well-being [35], and even social development [15, 36, 37]. Furthermore, feeding difficulties negatively influence parent–child interaction, resulting in lagged cognitive development and reduced emotional well-being [2, 4, 12, 20, 27, 28, 34].

To measure feeding difficulties from the oral motor perspective, a number of reliable and validated instruments have been developed [20, 21], such as the OSF and the SOMA. To objectify parents’ experiences, the MCH-FS has been suggested as a frontline screener for measuring such parentally reported feeding problems because it is very short and has strong psychometric properties [12]. The instrument contains a domain focusing on oral motor skills, which makes it comparable with the OSF and SOMA.

Regarding parent-reported feeding difficulties as measured by the MCH-FSD, we expected that children with CL/P would score higher, indicating more feeding problems, because facial clefts are known to influence the feeding process negatively [4–7]. However, the CL/P group showed significantly fewer feeding problems than the control group, though the differences were small. A possible explanation is that parents of children with CL/P, confronted with their situation, are possibly adjusted regarding the present feeding difficulties. As such, we suspect that these parents have an alternate frame of reference. In turn, this knowledge possibly leads to a deflation in their MCH-FSD score, as compared to parents with healthy children. A similar effect was also observed for parents of children with Down’s syndrome [38] and children who were born prematurely [39]. This suggests that although functional eating problems may occur more, caregivers generally do not report them as being more problematic.

In question 1b, the item analysis showed that there were significant differences between groups for items (1,2,3,5,13), where the CL/P group’s item scores were somewhat lower, in line with the result found in question 1a. Contrastingly, for item 11 which measures the parent’s perception of the child’s chewing and sucking ability, a higher score was observed, meaning that parents rate their CL/P child’s sucking and chewing skill to be lower than parents in the control group. An explanation for this is that the anatomical features of clefts inhibit the child from successfully creating suction because the oral cavity cannot be adequately separated from the nasal cavity during feeding [2, 14], which is also observed by parents. This result seems to confirm that parents can successfully detect these problems.

In this study, parentally reported feeding problems were compared to objective observations from a speech therapist on oral motor feeding skill. The main result was that MCH-FSD did not correlate with either the OSF or SOMA. The fact that no association between these constructs seems to exist suggests that parents of children with more severe oral motor problems do not necessarily report more feeding problems. It also shows that the full impact of feeding problems cannot be assessed by exclusively focusing on objective measures of feeding problems, because these are not necessarily related to the perspective of caregivers.

Furthermore, a correlation between the OSF and SOMA-solids and SOMA-cracker was found. This relationship was expected, considering the nature of the questions in both measuring instruments: while a high score in OSF indicates complete control of the skill “spoon feeding,” a low score on SOMA-solids and SOMA-cracker indicates normal functioning of oral motor skills. In fact, this observation can be used to confirm that CL/P children who are skilled at spoon feeding at an earlier age, also show oral skill with solid foods (e.g., fruit or cracker) at a later age. If this is not the case, then it might be a reason to invest in extra counseling on oral motor skills.

Regarding the limitations of this study, we must consider that the MCH-FSD scores that were obtained from the CL/P group were filled out in the presence of a researcher (in contrast to the control group questionnaires, which were filled out by parents alone). Furthermore, the item at which parents reported more problems also turned out to be unclear for the CL/P group because the item includes both sucking and chewing skills. These skills may be very different for children with CL/P. As many children were capable of chewing but not sucking, this resulted in varying answers based on the interpretation of the question. Also, despite the good psychometric characteristics of the OSF and SOMA, no measure of inter/intra-rater reliability was taken. Lastly, we did not transform the data that showed a non-normal distribution. Since the Box’ M did not differ significantly, a MANOVA was executed.

## Conclusion and practical implications

Early detection of feeding problems is essential due to the risk of serious complications. The MCH-FS is likely to be of great value in the early identification during a routine check-up of yet unknown parentally reported feeding difficulties, in addition to objective measures of feeding skills. The present study has revealed that there is no association between the MCH-FSD and the OSF and SOMA, which suggests that parents of children with more severe oral motor problems do not necessarily report more feeding problems than parents with healthy children. It is important to note that parental reporting and functional feeding limitations are two different dimensions of feeding behavior. This underscores the importance of combining both parental observations and measurements of oral motor skills because it allows healthcare professionals to objectively interpret and address parentally reported feeding problems at an early stage, relate them to the level of oral motor skills, and use both to enable adequate treatment of feeding difficulties in children with CL/P.

## Abbreviations

CL: Cleft lip
CL/P: Cleft lip and/or palate
CPO: Cleft of the palate only
IWF: Infant Welfare Centers
MCH-FS: Montreal Children’s Hospital Feeding Scale
MCH-FSD: Montreal Children’s Hospital Feeding Scale Dutch validated translation
OSF: Observation list spoon feeding
SOMA: Schedule for oral motor assessment
WCH: Wilhelmina Children’s Hospital
